# P-461. Positive Aging Consultations: A Model for Expanding Geriatric HIV Care

**DOI:** 10.1093/ofid/ofae631.660

**Published:** 2025-01-29

**Authors:** Sarah Gorvetzian, Kristine M Erlandson, Evelyn Iriarte, Jacob Walker

**Affiliations:** University of Colorado, Denver, Colorado; University of Colorado Anschutz Medical Campus, Aurora, CO; University of Colorado, Denver, Colorado; University of Colorado, Denver, Colorado

## Abstract

**Background:**

People living with HIV (PWH) experience geriatric syndromes commonly found in much older populations and may benefit from

geriatrician consultation. We aim to describe a referral-based model at the University of Colorado.

**Methods:**

The Positive Aging Consultations, a referral-based model, sought to connect high-risk PWH ≥ 50 years old with geriatric care. High-risk patients (experiencing geriatric syndromes, polypharmacy, multimorbidity, or advance care planning needs) were referred for geriatric consultation at the UCHealth Seniors Clinic; those taking ≥ 10 medications were referred for clinical pharmacy consultation. Patients were screened for functional status, preventative care, socioenvironmental factors (living situation, food access, transportation, social support, and elder abuse), and cognitive/mental health concerns (depression, anxiety, and memory loss). Geriatricians placed orders or communicated recommendations to the patient’s primary HIV provider. HIV providers completed a survey about their experience with the program. Additional data was collected via chart review.

**Results:**

From January 2018 to July 2019, 11 patients underwent geriatric consultation. The average age was 69 years old (*SD*=7; range 59-80). Geriatricians most often made recommendations regarding referral to other health or community-based services (*n*=9), medication changes (*n*=8), osteoporosis screening (*n*=6), and memory loss management (*n*=5). Advance directives were discussed in all visits. All but one HIV provider (*n*=7) said they would refer patients to the program in the future.

**Conclusion:**

The Positive Aging Consultations program improved access to geriatric care with minimal setup costs, but with limited uptake

over a one-year period. Limitations of the program included low utilization, lack of longitudinal geriatric care, geriatrician

shortage, and potential financial barriers.

**Disclosures:**

**Kristine M. Erlandson, MD MS**, Gilead: Advisor/Consultant|Gilead: Grant/Research Support|ViiV: Advisor/Consultant

